# Virological Mechanisms in the Coinfection between HIV and HCV

**DOI:** 10.1155/2015/320532

**Published:** 2015-10-01

**Authors:** Maria Carla Liberto, Emilia Zicca, Grazia Pavia, Angela Quirino, Nadia Marascio, Carlo Torti, Alfredo Focà

**Affiliations:** ^1^Department of Health Sciences, Institute of Microbiology, School of Medicine, University of “Magna Graecia”, Viale Europa, Germaneto, 88100 Catanzaro, Italy; ^2^Department of Medical and Surgical Sciences, Unit of Infectious and Tropical Diseases, School of Medicine, University of “Magna Graecia”, Viale Europa, Germaneto, 88100 Catanzaro, Italy

## Abstract

Due to shared transmission routes, coinfection with Hepatitis C Virus (HCV) is common in patients infected by Human Immunodeficiency Virus (HIV). The immune-pathogenesis of liver disease in HIV/HCV coinfected patients is a multifactorial process. Several studies demonstrated that HIV worsens the course of HCV infection, increasing the risk of cirrhosis and hepatocellular carcinoma. Also, HCV might increase immunological defects due to HIV and risk of comorbidities. A specific cross-talk among HIV and HCV proteins in coinfected patients modulates the natural history, the immune responses, and the life cycle of both viruses. These effects are mediated by immune mechanisms and by a cross-talk between the two viruses which could interfere with host defense mechanisms. In this review, we focus on some virological/immunological mechanisms of the pathogenetic interactions between HIV and HCV in the human host.

## 1. Introduction

Hepatitis C Virus (HCV) and Human Immunodeficiency Virus (HIV) cause considerable global health problems. Coinfection with HCV is frequent in HIV infected individuals, because the viruses share their modes of transmission. So, in the United States, approximately 25% of 1.2 million HIV infected patients are coinfected with HCV [1, 2]. Moreover, in Europe and Asia, rates of HCV coinfections among HIV infected individuals who used injection drugs overcame 90% [2]. Although in the era of Highly Active Antiretroviral Therapy (HAART) worthy achievements have been obtained in the treatment of HIV and HCV infections, HCV-related liver disease remains a significant therapeutic challenge in HIV/HCV coinfected patients.

While HCV is mainly a hepatotropic virus, HIV infects a variety of immune cells, such as CD4+ T lymphocytes and monocytes/macrophages. However, several studies showed that HCV replicates outside the liver [3], while HIV may infect hepatocytes and Hepatic Stellate Cells (HSCs) as well [4].

The disease course of HIV-1 infection is associated with a profound dysregulation of the immune system. During HIV/HCV coinfection, the immune dysregulation is more severe, leading to lower rates of spontaneous control of HCV infection as well as to a faster progression of liver disease [5]. The immunopathogenesis of accelerated hepatic fibrosis is a multifactorial event [6] and several mechanisms have been postulated:

1. HIV associated immune dysfunction;
2. defective antiviral CD8+ T cells responses;
3. reduced CD4/CD8 ratio;
4. direct activation of HSCs by HIV gp 120 or via proinflammatory mediators [7–9];
5. HIV and HCV inducing production of Reactive Oxygen Species (ROS) which activate the Mitogen-Activated Protein Kinases (MAPK) pathway and upregulate TGF-*β* [10, 11];
6. stimulation of HCV infected hepatocytes by HIV gp 120 that induces HCV replication via TGF-*β*, which modulates the immune response and favors fibrosis and transformation toward hepatocellular carcinoma [1, 10];
7. hepatocytes exposed to HCV and HIV envelope proteins that undergo apoptosis and, in particular, HCV E2 and HIV gp 120 that induce apoptosis via a Fas-FasL-dependent pathway [5–7];
8. HCV core and NS3 proteins that trigger, through Toll-Like Receptor- (TLR-) 2 and Interleukin- (IL-) 1 Receptor-Associated Kinase (IRAK) activity, the release of inflammatory cytokines and chemokines by HSCs [6].

Although the impact of HCV on HIV natural history is debated, the contribution of HCV core in enhancing HIV-1 infection in macrophages has been recently established [12]. Also, it has been suggested by some studies, but not by others, that the immunological response after HAART is impaired in HCV coinfected patients [13].

In this review we focus on the influence of HIV on HCV infection and vice versa. In particular, virological and immune escape mechanisms of HIV/HCV pathogenesis have been reviewed.

## 2. HIV/HCV Infection as Challenge to the Immune System

HCV and HIV evade the host immune response through intricate processes including signaling interference, effector modulation, and continuing viral genetic variations [1, 14].

Viral RNA is recognized via TLRs or the Retinoic Acid Inducible Gene I (RIG-I) helicase [15]. These interactions activate downstream signaling pathways, inducing type I interferon (IFN*α*/*β*) and other antiviral effects, which are the first innate immune response against intracellular pathogens [16].

IFN*α*/*β* is produced by plasmacytoid dendritic cells (pDCs), myeloid DCs (mDCs), and hepatocytes [16]. The production of IFN-*α* induces the expression of several IFN Regulatory Factors (IRFs) and the induction of IFN-Stimulated Genes (ISGs), leading to an antiviral state in cells and promotion of proliferation [17, 18].

HCV acts with several immune escape mechanisms interfering with type I interferon signal transduction molecules [16]. For instance, HCV NS3/4 protease cleaves proteins such as TIR-domain-containing adapter-inducing interferon-*β* (TRIF) and CARD adapter-inducing interferon-*β* (CARDIF), which are required for rapid induction of IFN-*β* through the activation of IRF-3 [19]. Moreover, NS5A, a protein that plays a crucial role in viral RNA replication, virus assembly, and viral pathogenesis [20], and E2, an envelope glycoprotein [21], mediate inhibition of protein kinase RNA-activated (PKR) activity [22].

Similarly, HIV-1 takes part in pathogen-associated molecular pattern via TLRs, because it contains motifs that are recognized by TLR8 [23] and TLR7 [1] and also interfere with PKR activity through Tat protein, a small activator of viral transcription from the Long Terminal Repeat (LTR) promoter [24].

Type I IFN secreted from DCs promotes recruitment and activation of natural killer (NK) cells to the site of infection [20].

NK cells are mediators of antiviral defenses and constitute a significant proportion of liver-infiltrating lymphocytes during HCV infection and an impairment of their function favors viral persistence [20].

In particular, HCV E2 protein can interfere with NK receptors, altering their function through cross-linking of CD81 [25, 26]. In fact, Tseng and Klimpel demonstrated that CD81 cross-linking inhibits cytotoxicity and IFN production of NK cells through immobilized E2 or specific CD81 monoclonal antibody (mAb) [27]. On the side of HIV, the following are well recognized: the decrease in number and dysregulation of CD4+ T cell function, the reduction of antifibrotic activity of NK cells, and production of an atypical* milieu* of ILs (IL-4, IL-5, IL-10, and IL-13) [25, 26]. Altogether these events may contribute to a profound dysregulation of the immune system as follows.

Dysregulation of the immune system in the pathogenesis of liver fibrosis progression (HIV/HCV coinfected patients) is as follows:

1. lymphocyte apoptosis and CD4+ T cell depletion [1, 28–30];
2. imbalance among CD4 and CD8 cell responses: this altered ratio is correlated with modified cytokine networks (such as increase in TGF-*β* and decrease in IFN-*γ* [6];
3. “by-stander effect”: HIV-1-specific CD8+ T cells are attracted to the liver of HIV/HCV coinfected patients, contributing to release of profibrotic cytokines [29];
4. aberrant dysregulation of natural killer cells function which leads to altered secretion of cytokines [25–27, 31–33].

Thus, a complex framework is established in the pathogenesis of HIV/HCV coinfections which ends up with a fast progression of liver disease [31].

## 3. Do HIV Proteins Affect HCV Infection?

Among HIV proteins, a role of gp 120, Rev, Tat, Nef, and Vpr in enhancing HCV replication has been established [10, 34–37].

HIV infection produces effects on hepatocytes and HSCs; indeed, both cells express key HIV coreceptors; the interaction of HIV gp 120 with C–C chemokine receptor type 5 (CCR5) and C–X–C chemokine receptor type 4 (CXCR4) activates specific cell signaling in the liver [6, 28, 29, 38, 39].

The HIV envelope protein gp 120 has been shown to promote hepatocyte apoptosis, hepatocellular secretion of the proinflammatory cytokine IL-8 [38], proinflammatory and profibrogenic effects on HSCs, and directional migration [9].

Moreover, a link between HIV infection and liver fibrogenesis has been demonstrated* in vitro*. In particular, HIV gp 120 induces a significant increase in HSCs chemotaxis and increased expression of the proinflammatory chemokine Monocyte Chemoattractant Protein-1 (MCP-1), IL-6, and tissue inhibitor of metalloproteinase-1 (TIMP-1), thereby leading to increased liver inflammation and fibrogenesis [9].

Concerning its effect on increased HCV replication, as demonstrated by Lin et al. [10], it has been shown that inactivated HIV and gp 120 enhance HCV replication in a CXCR4 or CCR5 engagement-dependent manner. Enhancements of HCV-regulated Transforming Growth Factor- (TGF-) *β*1 have been also shown in both a replicon and an infectious model of HCV infection [10].

Coinfection with HIV-1 causes increased HCV viral loads, as well as enhanced morbidity in coinfected individuals [40]. HIV contributes to the stimulation of HCV replication and this may change the course of HCV-related liver disease. Mechanisms of HIV effects on HCV replication are not fully clear. However, upregulation of HCV replication may be due to HIV viral proteins, which are secreted from HIV infected cells and diffused into hepatocytes [11, 34].

As demonstrated by Qu et al. [41], HIV Rev protein is a pivotal regulatory protein in the early-to-late switch in the virus life cycle and is involved in the promotion of translocation into the nuclear compartment, translation, and encapsidation of viral RNAs [41]. HIV Rev protein stimulates HCV gene expression through its binding with first internal loop (IIIb) of 5′-Untranslated Region (5′-UTR) HCV RNA [41]. Moreover, Rev regulates Internal Ribosome Entry Site- (IRES-) mediated translation of HCV* via* an enhanced polysomal association of Rev-responsive element- (RRE-) containing RNAs [42].

HIV-1 Tat is a transactivating protein which determines transactivation of viral and cellular genes [43], triggering virus invasion [44, 45]. Tat is released from HIV infected cells and induces its biological effects such as cytokine expression and CCR5 and CXCR4 receptors on neighboring infected or uninfected cells [46]. Qu et al. [35] used two different infectious HCV models to investigate the effects of HIV-1 Tat and interferon gamma-induced protein 10 (IP-10) on HCV replication, demonstrating that both HIV-1 Tat and IP-10 activate HCV replication. Moreover, HIV-1 Tat activates HCV replication by upregulation of IP-10 production, which in turn has been correlated with increased liver damage and higher HCV RNA in HIV/HCV coinfected patients [47].

The viral protein Nef exerts pleiotropic effects during HIV infection and regulates multiple host factors [48]. Nef modifies actin remodeling in various cell systems, alters actin rearrangements, and inhibits immunological synapse formation [49]. Nef also induces the extension of long intercellular conduits allowing its own transfer [50]. Moreover, as demonstrated by Park et al. [34], HIV Nef protein increases HCV replication in the target cells (such as subgenomic replicon cells) most likely through changes in the size and number of lipid droplets. Indeed, it is interesting to observe a drastic increase of HCV replication and an increase of ROS (a critical regulator of hepatic fibrosis progression) when the Nef-expressing cells are treated with ethanol [34, 51–53].

HIV Vpr, a multifunctional protein, mediates many processes such as activation of HIV-1 infection, evasion of the immune system, and induction of infection persistence in the host [54]. Vpr molecular functions include the following: (i) nuclear import of viral Preintegration Complex (PIC); (ii) transcriptional activation of viral and host genes; (iii) regulation of Nuclear Factor kappa B (NF-*κ*B) activity; and (iv) modulation of T-cell apoptosis [54]. Peng et al. [55] provided evidences to support relationships among HIV Vpr, microRNA 122 (miR-122), and HCV replication. However, several lines of evidence showed that Vpr promotes not only HCV RNA replication, but also protein expression, enhancing the HCV 5′ UTR activity through the stimulation of TATA box in the miR-122 promoter [55–58]. Moreover, miR-122 inhibition produces a significant reduction of Vpr-induced HCV 5′ UTR activity [55]. However, miR-122 inhibitor cannot fully abrogate the Vpr-induced HCV replication, suggesting that other mechanisms may create a favorable environment for maximizing virus replication [59, 60].

## 4. Do HCV Proteins Affect HIV Infection?

Despite the fact that HCV coinfection is able to increase immune activation and CD4 apoptosis [3], the mechanisms by which HCV modulates HIV replication are not completely understood.

HCV is a positive strand RNA virus whose genome encodes for a single polyprotein cleaved by host and cellular proteases to generate four structural (Core, E1, E2, and p7) and six nonstructural (NS2, NS3, NS4A, NS4B, NS5A, and NS5B) proteins [61].

The current knowledge on HCV proteins in the regulation of HIV-1 replication and the molecular mechanism involved will be reviewed below.

NS3 and NS4A proteins associate to form an active enzyme with RNA helicase and serine protease activities involved in the proteolytic processing of NS proteins. NS4A protein is required for proper functioning, while NS3 is a multifunctional protein with serine protease activity at the N-terminal and a Nucleoside-Triphosphatase (NTPase) dependent RNA-helicase activity (NS3 NTPase/helicase) at the C-terminal [62].

As demonstrated for the first time by Simmonds [61], the HCV NS3/4A protein can activate HIV-1 transcription from its LTR region, suggesting that binding activities of the transcription factor activating protein-1 (AP-1) are part of the mechanism involved. The serine protease activity of NS3/4A is essential for such activation effect [66]. Kang et al. developed an* in vitro* model of coinfection [36], showing that NS3 protein of HCV enhances HIV-1 LTR transcription and that Vpu protein regulates transcription of the HIV-1 genome by interacting with NS3/NS4A complex of HCV. Indeed, Vpu removes NS3 from the NS3/NS4A complex of HCV, thus promoting NS3 nuclear translocation for the activation of HIV-1 transcription [36].

HCV core is mainly a cytoplasmic component, located on the endoplasmic reticulum membranes and around lipid vesicles [67]. It is unique in its pleiotropic effects: in addition to its role in packaging viral RNA, it can indeed modulate cellular transduction pathways, transactivate a number of cellular promoters, regulate viral and cellular gene expression, modulate apoptosis, and inhibit host immunity [68].

Although contributions of HCV core to HIV pathogenesis remain controversial, this core protein has been shown to restrict HIV-1 transcription and modulate viral replication in a hepatoma cell line through a repression before accumulation of threshold levels of Tat protein [63]. Moreover, to better explain the influence of the HCV on HIV replication, Sengupta et al. [3] evaluated HIV LTR in hepatocytes through the analysis of basal and/or Tat-induced activation in presence of HCV core protein, TNF-*α*, and infectious HCV [3]. These authors demonstrated that HIV LTR activity was downregulated by HCV core protein with high TNF-*α* levels and that, conversely, it was increased by infectious hepatitis C virions. These data suggest that inhibitory activity of HCV core protein is unchanged and both host cellular where HCV viral proteins influence HIV replication [3].

Khan et al. [64] proposed that HIV-1 Nef and HCV core protein activate the NF-*κ*B canonical pathway in primary macrophages through TNF Receptor Associated Factor (TRAF) 2, TRAF5, and TRAF6 pathways and enhance HIV-1 LTR-driven luciferase expression in a transiently transfected human monocytic cell line through the same pathways. Therefore, this mechanism may promote HIV-1 replication and represent a critical factor for optimal replication of HIV-1 in macrophages of HIV-HCV-coinfected patients. Lastly, Swaminathan et al. [12] analyzed the effect of HCV core on HIV-1 replication in promonocytic cell line THP-1, primary monocyte-derived macrophages (MDMs), and in the HIV-1 latently infected U1 monocytic cell lines. They found that HCV core enhances HIV-1 infection in both THP-1 cells and primary macrophages. Particularly, HCV core protein promotes HIV-1 infectivity in macrophages via TLR2-, JNK-, and MEK1/2-dependent pathways, while a differential activation/regulation of p38 kinase in THP-1 and MDMs was found. Interestingly, although HCV core lacks ability to directly reactivate HIV-1 in latently infected U1 cells, conditioned media (CM) of THP-1 macrophages and primary MDMs of HCV core-stimulated macrophages induced HIV-1 reactivation in U1 cells through TNF-*α* and IL-6. Therefore, these studies definitely established a role of HCV core in exacerbation of acute and latent HIV-1 infection in macrophages.

Interactions between HIV and HCV proteins and their effect on replication of both viruses are summarized in Table 1.

## 5. Conclusion

Several* in vitro* and* in vivo* studies indicated that HCV and HIV interact with each other and with innate or adaptive immune system exerting a variety of effects and promoting a series of hypothesis to be tested in future studies as follows:

1. HIV infection is associated with a profound dysregulation of the immune system; during HIV/HCV coinfection, this immune dysregulation is more severe, leading to lower rates of spontaneous control of HCV replication and to a faster progression of liver disease [5, 6];
2. cross-talk among HCV and HIV proteins modulates fibrogenic/inflammatory mediators, immune system response, and replication of both viruses [6];
3. characterization of viral protein interactions and their effects both on replication of these viruses and on liver function at a cellular level will significantly improve our understanding of HIV/HCV pathogenesis [3, 34, 36, 55, 59, 64, 66].

It is clear from this review that several problems remain to be understood for a better comprehension of the multiple virus-virus and virus-host interactions that can lead to liver fibrosis and enhancement of the pathogenetic effects of both viruses. The development of coculture systems that model the effects of HCV/HIV in hepatocytes will advance our understanding of the pathogenesis of this coinfection. However, it is not obvious that* in vitro* interactions are confirmed* in vivo*. So, a suitable animal model could provide deeper understanding of virus-virus interactions and immunological relationships. Hopefully, recognition of the cause-effect relationships between infection, inflammation, and liver fibrosis progression in HIV/HCV coinfected patients could lead to therapeutic approaches to better control these viruses.

## Figures and Tables

**Table 1:** Interactions among proteins of HIV and HCV (relevance for HCV/HIV coinfection models).

HCV proteins	Effect on HIV replication
NS3/NS4A	It interacts with HIV-1 Vpu promoting HIV transcription. Vpu facilitates degradation of NS3/4A and nuclear transfer of NS3 which can activate HIV-1 transcription [36].
Core	It restricts HIV-1 transcription and modulates viral replication in a hepatoma cell line through a repression before accumulation of threshold levels of Tat protein [63].
	It downregulates HIV LTR activity, in presence of high TNF-*α* level [3].
	It activates the TRAF pathway interacting with HIV-1 Nef, activating the NF-*κ*B pathway via TRAF2, TRAF5, and TRAF6 pathways, and enhancing HIV-1 replication in macrophages [64].
	It induces HIV-1 reactivation in U1 cells through TNF-*α* and IL-6 [12].

HIV proteins	Effect on HCV replication

gp120	It enhances HCV replication in a CXCR4 or CCR5 engagement-dependent manner [9–11, 31, 34–36, 38–40, 65].
Rev	It increases gene expression of HCV by binding to the first internal loop (IIIb) of 5′-Untranslated Region and sites IRES of HCV RNA [42].
Tat	It activates HCV replication by upregulating IP-10 [47].
Nef	It exerts stimulatory effects on HCV replication, modifying the size and numbers of lipid droplets, increasing ROS, and, possibly, accelerating progression of liver disease [51–53].
Vpr	It enhances activity of 5′-Untranslated Region of HCV through stimulation of TATA box in the miR-122 promoter, upregulating miR-122 expression [55, 59, 60].

HCV: Hepatitis C Virus; HIV-1: Human Immunodeficiency Virus 1; NS: Nonstructural protein; CXCR4: C-X-C chemokine receptor type 4; CCR5: C-C chemokine receptor type 5; IRES: Internal Ribosome Entry Site; LTR: Long Terminal Repeat; TNF-*α*: Tumor Necrosis Factor alfa; IP-10: interferon gamma-induced protein 10; TRAF: TNF Receptor Associated Factor; NF-*κ*B: Nuclear Factor *κ*B; ROS: Reactive Oxygen Species; U1: HIV-1 latently infected U1 monocytic cell line; IL: Interleukin; miR: microRNA.

## References

[1] Kim A. Y., Chung R. T. (2009). Coinfection with HIV-1 and HCV—A one-two punch. *Gastroenterology*.

[2] Chen J. Y., Feeney E. R., Chung R. T. (2014). HCV and HIV co-infection: mechanisms and management. *Nature Reviews Gastroenterology and Hepatology*.

[3] Sengupta S., Powell E., Kong L., Blackard J. T. (2013). Effects of HCV on basal and Tat-induced HIV LTR activation. *PLoS ONE*.

[4] Kong L., Maya W. C., Moreno-Fernandez M. E. (2012). Low-level HIV infection of hepatocytes. *Virology Journal*.

[5] Ansari A. W., Schmidt R. E., Shankar E. M., Kamarulzaman A. (2014). Immuno-pathomechanism of liver fibrosis: targeting chemokine CCL2-mediated HIV:HCV nexus. *Journal of Translational Medicine*.

[6] Mastroianni C. M., Lichtner M., Mascia C., Zuccalà P., Vullo V. (2014). Molecular mechanisms of liver fibrosis in HIV/HCV coinfection. *International Journal of Molecular Sciences*.

[7] Lin W., Weinberg E. M., Chung R. T. (2013). Pathogenesis of accelerated fibrosis in HIV/HCV co-infection. *Journal of Infectious Diseases*.

[8] Friedman S. L. (2008). Mechanisms of hepatic fibrogenesis. *Gastroenterology*.

[9] Bruno R., Galastri S., Sacchi P. (2010). gp120 modulates the biology of human hepatic stellate cells: a link between HIV infection and liver fibrogenesis. *Gut*.

[10] Lin W., Weinberg E. M., Tai A. W. (2008). HIV increases HCV replication in a TGF-beta1-dependent manner. *Gastroenterology*.

[11] Lin W., Tsai W.-L., Shao R.-X. (2010). Hepatitis C virus regulates transforming growth factor beta1 production through the generation of reactive oxygen species in a nuclear factor kappaB-dependent manner. *Gastroenterology*.

[12] Swaminathan G., Pascual D., Rival G., Perales-Linares R., Martin-Garcia J., Navas-Martin S. (2014). Hepatitis C virus core protein enhances HIV-1 replication in human macrophages through TLR2, JNK, and MEK1/2-dependent upregulation of TNF-*α* and IL-6. *FEBS Letters*.

[13] Tsiara C. G., Nikolopoulos G. K., Dimou N. L. (2013). Effect of hepatitis C virus on immunological and virological responses in HIV-infected patients initiating highly active antiretroviral therapy: a meta-analysis. *Journal of Viral Hepatitis*.

[14] Gale M., Foy E. M. (2005). Evasion of intracellular host defence by hepatitis C virus. *Nature*.

[15] Yang D. R., Zhu H. Z. (2015). Hepatitis C virus and antiviral innate immunity: who wins at tug-of-war?. *World Journal of Gastroenterology*.

[16] Gonzalez V. D., Landay A. L., Sandberg J. K. (2010). Innate immunity and chronic immune activation in HCV/HIV-1 co-infection. *Clinical Immunology*.

[17] Gerosa F., Baldani-Guerra B., Nisii C., Marchesini V., Carra G., Trinchieri G. (2002). Reciprocal activating interaction between natural killer cells and dendritic cells. *Journal of Experimental Medicine*.

[18] Megjugorac N. J., Young H. A., Amrute S. B., Olshalsky S. L., Fitzgerald-Bocarsly P. (2004). Virally stimulated plasmacytoid dendritic cells produce chemokines and induce migration of T and NK cells. *Journal of Leukocyte Biology*.

[19] Sianipar I. R., Matsui C., Minami N. (2015). Physical and functional interaction between hepatitis C virus NS5A protein and ovarian tumor protein deubiquitinase 7B. *Microbiology and Immunology*.

[20] Dansako H., Ikeda M., Kato N. (2007). Limited suppression of the interferon-*β* production by hepatitis C virus serine protease in cultured human hepatocytes. *The FEBS Journal*.

[21] Falson P., Bartosch B., Alsaleh K. (2015). Hepatitis C virus envelope glycoprotein E1 forms trimers at the surface of the virion. *Journal of Virology*.

[22] Xiang J., Martinez-Smith C., Gale M. (2005). GB virus type C NS5A sequence polymorphisms: association with interferon susceptibility and inhibition of PKR-mediated eIF2alpha phosphorylation. *Journal of Interferon and Cytokine Research*.

[23] Chattergoon M. A., Latanich R., Quinn J. (2014). HIV and HCV activate the inflammasome in monocytes and macrophages via endosomal Toll-like receptors without induction of type 1. *PLoS Pathogens*.

[24] Yoon C. H., Kim S. Y., Byeon S. E. (2015). p53-derived host restriction of HIV-1 replication by protein kinase R-mediated tat phosphorylation and inactivation. *Journal of Virology*.

[25] Glässner A., Eisenhardt M., Kokordelis P. (2013). Impaired CD4^+^ T cell stimulation of NK cell anti-fibrotic activity may contribute to accelerated liver fibrosis progression in HIV/HCV patients. *Journal of Hepatology*.

[26] Mehal W. Z., Friedman S. L. (2007). The role of inflammation and immunity in the pathogenesis of liver fibrosis. *Liver Immunology*.

[27] Tseng C.-T. K., Klimpel G. R. (2002). Binding of the hepatitis C virus envelope protein E2 to CD81 inhibits natural killer cell functions. *Journal of Experimental Medicine*.

[28] Vlahakis S. R., Villasis-Keever A., Gomez T. S., Bren G. D., Paya C. V. (2003). Human immunodeficiency virus-induced apoptosis of human hepatocytes via CXCR4. *Journal of Infectious Diseases*.

[29] Munshi N., Balasubramanian A., Koziel M., Ganju R. K., Groopman J. E. (2003). Hepatitis C and human immunodeficiency virus envelope proteins cooperatively induce hepatocytic apoptosis via an innocent bystander mechanism. *Journal of Infectious Diseases*.

[30] Balasubramanian A., Ganju R. K., Groopman J. E. (2006). Signal transducer and activator of transcription factor 1 mediates apoptosis induced by hepatitis C virus and HIV envelope proteins in hepatocytes. *Journal of Infectious Diseases*.

[31] Rotman Y., Liang T. J. (2009). Coinfection with hepatitis C virus and human immunodeficiency virus: virological, immunological, and clinical outcomes. *Journal of Virology*.

[32] Zhang S., Saha B., Kodys K., Szabo G. (2013). IFN-*γ* production by human natural killer cells in response to HCV-infected hepatoma cells is dependent on accessory cells. *Journal of Hepatology*.

[33] Saïdi H., Melki M.-T., Gougeon M.-L. (2008). HMGB1-dependent triggering of HIV-1 replication and persistence in dendritic cells as a consequence of NK-DC cross-talk. *PLoS ONE*.

[34] Park I.-W., Fan Y., Luo X. (2014). HIV-1 Nef is transferred from expressing T cells to hepatocytic cells through conduits and enhances HCV replication. *PLoS ONE*.

[35] Qu J., Zhang Q., Li Y. (2012). The Tat protein of human immunodeficiency virus-1 enhances hepatitis C virus replication through interferon gamma-inducible protein-10. *BMC Immunology*.

[36] Kang L., Luo Z., Li Y. (2012). Association of Vpu with hepatitis C virus NS3/4A stimulates transcription of type 1 human immunodeficiency virus. *Virus Research*.

[37] Deng A., Chen C., Ishizaka Y., Chen X., Sun B., Yang R. (2014). Human immunodeficiency virus type 1 Vpr increases hepatitis C virus RNA replication in cell culture. *Virus Research*.

[38] Balasubramanian A., Ganju R. K., Groopman J. E. (2003). Hepatitis C virus and HIV envelope proteins collaboratively mediate interleukin-8 secretion through activation of p38 MAP kinase and SHP2 in hepatocytes. *The Journal of Biological Chemistry*.

[39] Yoong K. E., Afford S. C., Jones R. (1999). Expression and function of CXC and CC chemokines in human malignant liver tumors: a role for human monokine induced by *γ*-interferon in lymphocyte recruitment to hepatocellular carcinoma. *Hepatology*.

[40] Bonacini M., Louie S., Bzowej N., Wohl A. R. (2004). Survival in patients with HIV infection and viral hepatitis B or C: a cohort study. *AIDS*.

[41] Qu J., Yang Z., Zhang Q. (2011). Human immunodeficiency virus-1 Rev protein activates hepatitis C virus gene expression by directly targeting the HCV 5′-untranslated region. *FEBS Letters*.

[42] D'Agostino D. M., Felber B. K., Harrison J. E., Pavlakis G. N. (1992). The Rev protein of human immunodeficiency virus type 1 promotes polysomal association and translation of gag/pol and vpu/env mRNAs. *Molecular and Cellular Biology*.

[43] Dayton A. I., Sodroski J. G., Rosen C. A., Goh W. C., Haseltine W. A. (1986). The *trans*-activator gene of the human T cell lymphotropic virus type III is required for replication. *Cell*.

[44] Stettner M. R., Nance J. A., Wright C. A. (2009). SMAD proteins of oligodendroglial cells regulate transcription of JC virus early and late genes coordinately with the Tat protein of human immunodeficiency virus type 1. *Journal of General Virology*.

[45] Nyagol J., Leucci E., Onnis A. (2006). The effects of HIV-1 Tat protein on cell cycle during cervical carcinogenesis. *Cancer Biology and Therapy*.

[46] Huang L., Bosch I., Hofmann W., Sodroski J., Pardee A. B. (1998). Tat protein induces human immunodeficiency virus type 1 (HIV-1) coreceptors and promotes infection with both macrophage-tropic and T-lymphotropic HIV-1 strains. *Journal of Virology*.

[47] Romero A. I., Lagging M., Westin J. (2006). Interferon (IFN)-*γ*-inducible protein-10: association with histological results, viral kinetics, and outcome during treatment with pegylated IFN-*α* 2a and ribavirin for chronic hepatitis C virus infection. *Journal of Infectious Diseases*.

[48] Joseph A. M., Kumar M., Mitra D. (2005). Nef: ‘necessary and enforcing factor’ in HIV infection. *Current HIV Research*.

[49] Thoulouze M. I., Sol-Foulon N., Blanchet F., Dautry-Varsat A., Schwartz O., Alcover A. (2006). Human immunodeficiency virus type-1 infection impairs the formation of the immunological synapse. *Immunity*.

[50] Nobile C., Rudnicka D., Hasan M. (2010). HIV-1 Nef inhibits ruffles, induces filopodia, and modulates migration of infected lymphocytes. *Journal of Virology*.

[51] Ivanov A. V., Bartosch B., Smirnova O. A., Isaguliants M. G., Kochetkov S. N. (2013). HCV and oxidative stress in the liver. *Viruses*.

[52] Ming-Ju H., Yih-Shou H., Tzy-Yen C., Hui-Ling C. (2011). Hepatitis C virus E2 protein induce reactive oxygen species (ROS)-related fibrogenesis in the HSC-T6 hepatic stellate cell line. *Journal of Cellular Biochemistry*.

[53] Cubero F. J., Nieto N. (2008). Ethanol and arachidonic acid synergize to activate Kupffer cells and modulate the fibrogenic response via tumor necrosis factor *α*, reduced glutathione, and transforming growth factor *β*–dependent mechanisms. *Hepatology*.

[54] Kogan M., Rappaport J. (2011). HIV-1 accessory protein Vpr: relevance in the pathogenesis of HIV and potential for therapeutic intervention. *Retrovirology*.

[55] Peng M., Xiao X., He Y. (2015). HIV Vpr protein upregulates microRNA-122 expression and stimulates hepatitis C virus replication. *Journal of General Virology*.

[56] Conrad K. D., Giering F., Erfurth C. (2013). MicroRNA-122 dependent binding of Ago2 protein to hepatitis C virus RNA is associated with enhanced RNA stability and translation stimulation. *PLoS ONE*.

[57] Henke J. I., Goergen D., Zheng J. (2008). microRNA-122 stimulates translation of hepatitis C virus RNA. *The EMBO Journal*.

[58] Shimakami T., Yamane D., Jangra R. K. (2012). Stabilization of hepatitis C virus RNA by an Ago2-miR-122 complex. *Proceedings of the National Academy of Sciences of the United States of America*.

[59] Goh W. C., Rogel M. E., Matthew Kinsey C. (1998). HIV-1 Vpr increases viral expression by manipulation of the cell cycle: a mechanism for selection of Vpr in vivo. *Nature Medicine*.

[60] Poon B., Grovit-Ferbas K., Stewart S. A., Chen I. S. Y. (1998). Cell cycle arrest by Vpr in HIV-1 virions and insensitivity to antiretroviral agents. *Science*.

[61] Simmonds P. (2013). The origin of hepatitis C virus. *Current Topics in Microbiology and Immunology*.

[62] Tai C.-L., Chi W.-K., Chen D.-S., Hwang L.-H. (1996). The helicase activity associated with hepatitis C virus nonstructural protein 3 (NS3). *Journal of Virology*.

[63] Srinivas R. V., Ray R. B., Meyer K., Ray R. (1996). Hepatitis C virus core protein inhibits human immunodeficiency virus type 1 replication. *Virus Research*.

[64] Khan K. A., Abbas W., Varin A. (2013). HIV-1 Nef interacts with HCV core, recruits TRAF2, TRAF5 and TRAF6, and stimulates HIV-1 replication in macrophages. *Journal of Innate Immunity*.

[65] Bobbin M. L., Burnett J. C., Rossi J. J. (2015). RNA interference approaches for treatment of HIV-1 infection. *Genome Medicine*.

[66] Wu X., Ishaq M., Hu J., Guo D. (2008). HCV NS3/4A protein activates HIV-1 transcription from its long terminal repeat. *Virus Research*.

[67] Bartosch B., Dubuisson J. (2010). Recent advances in hepatitis C virus cell entry. *Viruses*.

[68] Giannini C., Bréchot C. (2003). Hepatitis C virus biology. *Cell Death and Differentiation*.

